# Geometry, Electronic Structure, and Pseudo Jahn-Teller Effect in Tetrasilacyclobutadiene Analogues

**DOI:** 10.1038/srep23315

**Published:** 2016-03-21

**Authors:** Yang Liu, Ya Wang, Isaac B. Bersuker

**Affiliations:** 1 Academy of Fundamental and Interdisciplinary Sciences, Harbin Institute of Technology, Harbin, 150080, People’s Republic of China; 2 Institute for Theoretical Chemistry, University of Texas at Austin, Austin, TX 78712, USA

## Abstract

We revealed the origin of the structural features of a series of tetrasilacyclobutadiene analogues based on a detailed study of their electronic structure and the pseudo Jahn-Teller effect (PJTE). Starting with the D_4h_ symmetry of the Si_4_R_4_ system with a square four-membered silicon ring as a reference geometry, and employing ab initio calculations of energy profiles along lower-symmetry nuclear displacements in the ground and several excited states, we show that the ground-state boat-like and chair-like equilibrium configurations are produced by the PJT interaction with appropriate excited sates. For Si_4_F_4_ a full two-mode *b*_1g_−*b*_2g_ adiabatic potential energy surface is calculated showing explicitly the way of transformation from the unstable D_4h_ geometry to the two equilibrium C_2h_ configurations via the D_2h_ saddle point. The PJTE origin of these structural features is confirmed also by estimates of the vibronic coupling parameters. For Si_4_R_4_ with large substituents the origin of their structure is revealed by analyzing the PJT interaction between the frontier molecular orbitals. The preferred chair-like structures of Si_4_R_4_ analogues with amido substituents, and heavier germanium-containing systems Ge_4_R_4_ (potential precursors for semiconducting materials) are predicted.

Cyclobutadiene and its derivatives have attracted extensive attention of researchers for many years^1^,^2^,^3^,^4^,^5^,^6^,^7^,^8^,^9^. Among all kinds of its derivatives tetrasilacyclobutadiene analogues (denoted as Si_4_R_4_ hereafter) are the hottest topics due to the rich silicon chemistry and expected novel applications as semiconducting materials^1^,^5^,^10^. Recently, the interest in these compounds was enhanced by the synthesis of Si_4_(EMind)_4_ (EMind =1,1,7,7-tetraethyl-3,3,5,5-tetramethyl-s-hydrindacen-4-yl), and its structural characterization with a distinctive planar-rhombic four-member silicon ring^11^,^12^ (Fig. 1). Consequently, some new tetrasilacyclobutadiene or tetragermacyclobutadiene analogues that contain a planar-rhombic or puckered Si_4_ ring^13^,^14^, a slightly folded Ge_4_ ring^15^, or a puckered Si_3_Ge ring with ylide structure^16^ were synthesized. As the simplest member of Si_4_R_4_ family, the parent Si_4_H_4_ was predicted theoretically to have a puckered Si_4_ ring with D_2d_ symmetry^2^,^5^. From the simplest Si_4_H_4_ to the complicated Si_4_(EMind)_4_ compound the central silicon skeleton undergoes several low-symmetry configuration changes of the reference square-planar geometry.

The origin of this variety of the molecular geometries in the Si_4_R_4_ and Ge_4_R_4_ series can be rationalized by employing the vibronic coupling theory in the form of the pseudo Jahn-Teller effect (PJTE)^17^,^18^. This statement follows from a more general conclusion that the Jahn-Teller effect for degenerate electronic states and the PJTE for both degenerate and nondegenerate (pseudodegenerate) states are the only source of spontaneous symmetry breaking in molecular systems and solids (see references in refs 17, 18, 19). In our case of nondegenerate electronic states the PJTE provides a reasonable picture of the structure and properties of the compounds under consideration. The method has been successfully applied to more simple carbon and silicon four-membered ring systems, such as C_4_H_4_^20^, C_4_F_4_^7^, Si_4_^21^, Si_4_H_4_^2+ ^22, as well as to a variety of other molecular systems (see^18^,^23^,^24^,^25^,^26^,^27^,^28^,^29^,^30^ and references therein).

In this paper we report the results of a detailed analysis of the origin of the structural features of a series tetrasilacyclobutadiene analogues, Si_4_R_4_, including relatively large substituents R, as well as some other related systems, including Ge_4_R_4_, based on their electronic structure and vibronic coupling. Applying the PJTE theory, we reveal the lowest excited states that cause the distortion (puckering) of the high-symmetry planar configuration and estimate the vibronic coupling constants that control these distortions. This information provides also some clues for manipulation of the structure by means of external perturbations or substitutions, similar to the recently suggested methods of restoring planarity in puckered hexa- and tetra-heterocyclic systems^31^,^32^,^33^, thus inspiring the search of new materials with desired properties, in particular, other kinds of stable tetrasilacyclobutadiene analogues. Our theoretical prediction of stable structures of Si_4_R_4_ analogues with different substituents and Ge_4_R_4_ with bulky substituents is expected to provide also information for synthesizing new silicon or germanium four-member ring compounds and exploring their potential applications as semiconducting materials.

## Results and Discussion

### PJTE in the origin of Si_4_R_4_ geometries

**Figure 2:**
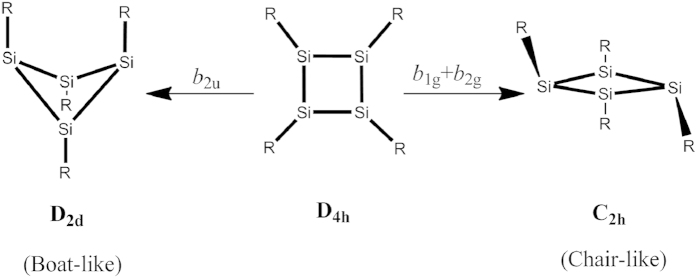
Two types of distortions of the highest-symmetry D_4h_ configuration of Si_4_R_4_ compounds induced by the PJTE: boat-like with D_2d_ and chair-like with C_2h_ symmetry.

There are two typical equilibrium structures of Si_4_R_4_ analogues, the one with a puckered Si_4_ ring as in Si_4_H_4_, which hereafter is denoted as a “boat-like” structure, and the other one with a planar-rhombic Si_4_ ring and alternating pyramidal and planar bonded substituents at the silicon atoms, which is denoted as “chair-like” structure hereafter (Fig. 2). To rationalize the origin of these boat-like and chair-like structures of Si_4_R_4_ analogues we start with one of the simpler representatives, the Si_4_F_4_ molecule; it allows revealing the main mechanisms of formation of boat-like and chair-like structures that are similar for all the Si_4_R_4_ systems.

**Figure 3:**
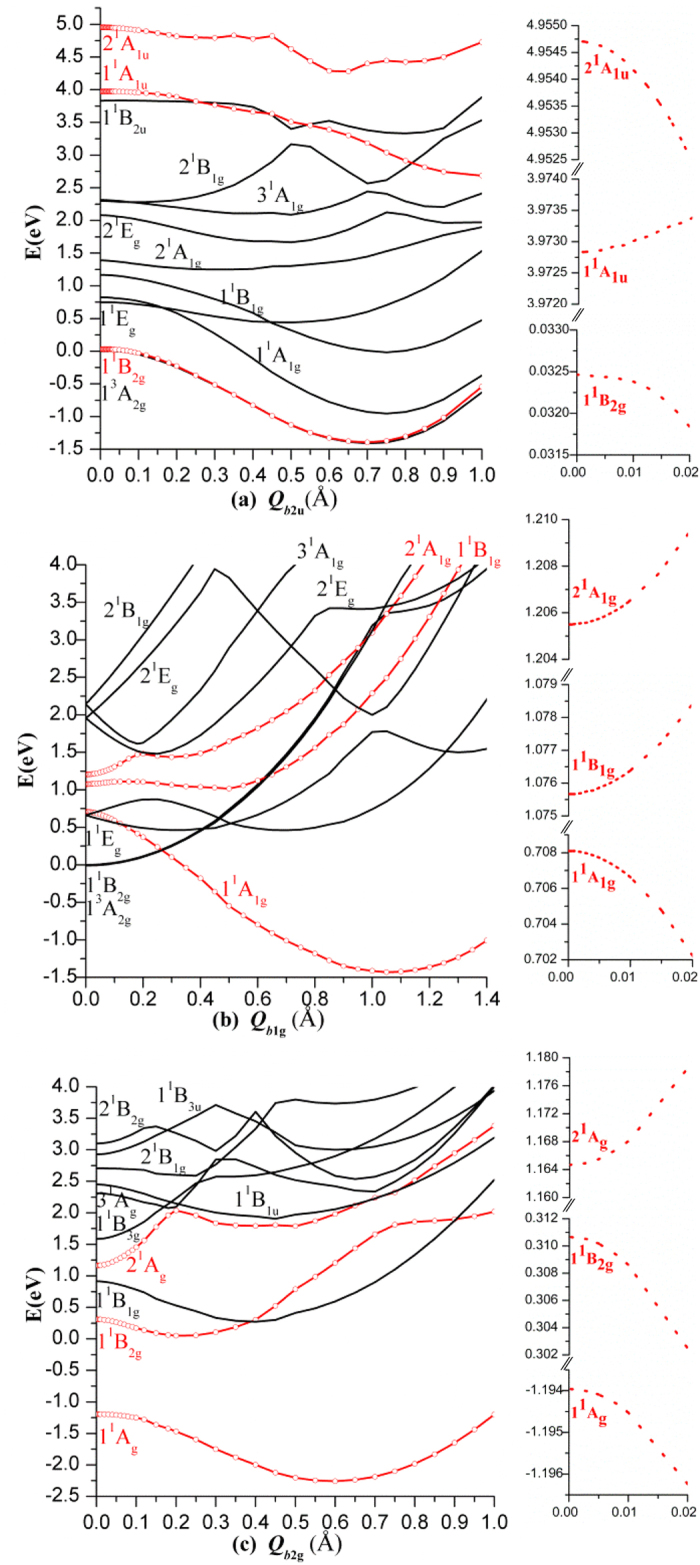
The potential energy profiles of the ground and low-lying excited states of the Si_4_F_4_ molecule along *b*_2u_ (**a**), *b*_1g_ (**b**) and *b*_2g_ (**c**) distortions by taking the energy of the D_4h_ configuration as the zero point. The lines in red with dots represent the coupling states, shown also in the inserts at small displacements (some states between ^1^A_1u_ and 2^1^A_1u_ in Fig. 3(a) are not shown, see Supplementary Fig. S2). The distortions along *b*_2g_ in Fig. 3(c) are conveniently started at Q_*b*1g_ = 0.74Å of Fig. 3(b), from which point there is a direct pass to the global minima shown in Fig. 4.

According to the general procedure, the PJTE formulation in this case starts from the highest symmetry D_4h_ configuration at which the molecule is square-planar with the central Si_4_ ring forming a square and the four ligands symmetrically bonded. Its transformations to the lower symmetry configurations is realized by symmetrized *b*_2u_ type displacements toward the boat-like geometry and via combined *b*_1g_ + *b*_2g_ displacements toward the chair-like configuration (Fig. 2). [Hereafter we employ small letters to denote the symmetry representations of both vibrational modes and molecular orbitals, and capital letters for electronic states]. Since the ground state in D_4h_ geometry is nondegenerate, according to the general theorem^17^,^18^ the instabilities are induced by the pseudo Jahn-Teller (PJT) coupling to appropriate excited electronic states. To reveal the latter we calculate and analyze the energy profiles (the cross section of the adiabatic potential energy surface (APES)) of the electronic ground and low-lying excited states along these normal displacements. The results are shown in Fig. 3.

Consider first the instability along the *b*_2u_ coordinate Q_*b*2u_ of molecular puckering that leads to the boat-like structure with D_2d_ symmetry. Note that Q denotes normal coordinates, not atomic displacements. As seen from Fig. 3(a), along *b*_2u_ the D_4h_ configuration in the singlet ground state 1^1^B_2g_ is unstable and distorts spontaneously up to the minimum point at Q = 0.7Å which is the boat-like structure of Si_4_F_4_ of Fig. 2 (the role of the triplet state is outlined below). According to the group theory rules the lowest excited states that couple with 1^1^B_2g_ via the *b*_2u_ mode in the D_4h_ symmetry are the 1^1^A_1u_ and 2^1^A_1u_ states, realizing the 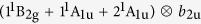 PJTE problem (the role of higher excited states is discussed in next section). Hence the formation of the boat-like structure in this system is due to the vibronic coupling with the excited ^1^A_1u_ states. From the estimated vibronic coupling constants below it follows that the main contribution comes from the 1^1^A_1u_ state. By comparing the electronic structures we can see that the 1^1^B_2g_ state comes from the *e*_g_^2^ electronic configuration, while the 1^1^A_1u_ state emerges from the electronic excitations of an *e*_g_ electron to the empty *e*_u_ orbital. The latter thus play an important role in the formation of boat-like structures.

**Figure 4:**
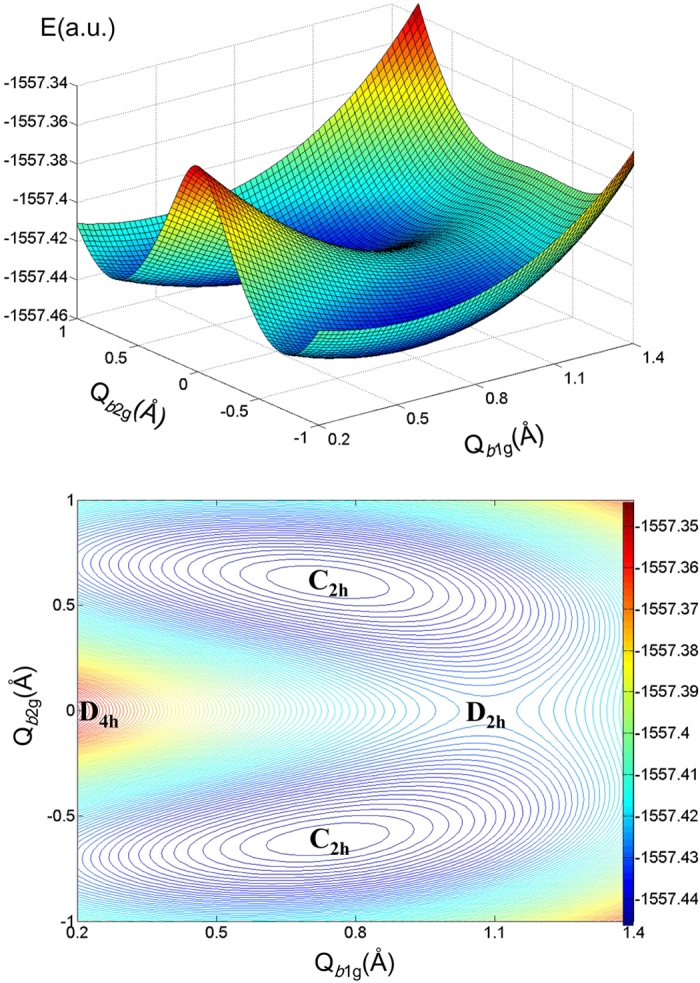
Calculated two-dimensional segment of the APES of the Si_4_F_4_ molecule (top) and its contour map (bottom) in the rhombic *b*_1g_ and puckering *b*_2g_ distortion coordinates.

The analysis of possible distortions along *b*_1g_ and *b*_2g_ is more complicated. From Fig. 3(b) we see that there is a hidden PJTE (meaning a sufficiently strong PJT interaction between two or more excited states that produces an additional global minimum with a distorted configuration, see ref. 29 for details): the PJT mixing of the three close excited states, 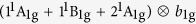, produces an additional potential minimum at Q_*b*1g_ = 1.1Å in which the Si_4_ framework is rhombically distorted. On the other hand, the ground state ^1^A_g_ is unstable with respect to the *b*_2g_ puckering, and it is a priori unclear where the global minimum might occur. This prompted further calculations of the two-dimensional part of the APES along two coordinates *b*_1g_ and *b*_2g_. The results are illustrated in Fig. 4. We see that, indeed, the minimum in the energy profile along *b*_1g_ is just a saddle point, from which the system descends along *b*_2g_ to the global minima of C_2h_ symmetry with a chair-like structure of Fig. 2. The normal coordinates (Q_b1g_, Q_b2g_) of these two minima (read off the D_4h_ point) in Å are about (0.74, ± 0.62). To reveal the excited states involved in these PJTE distortions we calculated the energy profiles of the system along *b*_2g_ (shown in Fig. 3(c)) beginning from the point Q_*b*1g_ = 0.74 Å of Fig. 3(b).

The results shown in Fig. 4 together with the energy profiles in Fig. 3 illustrate the full picture of instabilities and distorted equilibrium-geometry formation of the Si_4_F_4_ molecule. In the highest symmetry configuration D_4h_ the system is unstable along *b*_1g_ (rhombic distortion of the Si_4_ ring) due to the hidden PJTE problem 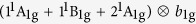 mixing two excited electronic states, followed by the puckering distortion *b*_2g_ (emerging from the 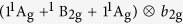 problem), resulting in an equilibrium chair-like structure at the minima of the APES (Fig. 2). On the other hand, as shown above, the APES of this system has another minimum along the *b*_2u_ displacements producing the boat-like equilibrium structure. However, because the two excited states 1^1^A_1g_ and 1^1^B_1g_ in the D_4h_ geometry are relatively very close, the hidden PJTE of their mixing along *b*_1g_ is much stronger than that of the 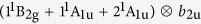 problem, so the chair-like structure is lower in energy by 0.39 eV than the boat-like structure. The fully optimized chair-like and boat-like Si_4_F_4_ geometrical coordinates are given in the Supplementary data S1. By examining the 1^1^B_1g_ excited state in the ab initio calculations we can see that dominant electronic configurations are produced by the electronic transitions from the occupied *e*_g_ to *b*_1g_ or *a*_1g_ empty molecular orbitals. The significant role of *b*_1g_ orbital in these interactions is discussed below in more detail.

Note that the triplet ^3^A_2g_ ground state in the D_4h_ geometry is not significant with respect to observable structural features of Si_4_F_4_ because it is stable with respect to *b*_1g_ distortions that lead to the global minima with a singlet electronic state (Fig. 3(b)), at which point ^3^A_2g_ is an excited state; its instability in D_4h_ along the *b*_2u_ mode leads to approximately the same boat-like minimum as in the singlet 1^1^B_2g_ state (Fig. 3(a)) which, as shown above, is much higher in energy than the chair-like minimum.

### Numerical estimation of the PJTE coupling parameters

The qualitative picture, which reveals the excited electronic states producing the instability of the ground state, obtained in the previous section, can be enhanced quantitatively by estimates of the numerical values of the vibronic coupling parameters in the PJTE. First of all such numerical estimates are important to limit the number of excited states that produce significant contribution to the instability in a given direction. Indeed, in any polyatomic system the number of excited states is practically infinite, and for any given low-symmetry displacement *Q* there are always some excited states of relevant symmetry that contribute to the destabilization of the ground state. In general, this contribution is rather small, just lowering the curvature of the ground state from *K*_0_ to *K*_0_−*p, p* > 0, and not producing instability, meaning *K*_0_ − *p* > 0, (according to the general theory^17^ the primary force constant without the vibronic coupling, *K*_0_ > 0), and only low-lying excited states with sufficiently large contribution to the PJT destabilization of the ground state may be the reason of instability, and only in certain directions *Q*.

Assuming that there is only one such excited state producing instability in the *Q* direction, we come to the two-level PJTE problem. The primary force constants in the ground and the active excited state is denoted by *K*_1_ and *K*_2_, respectively, and the second order perturbation corrections to them from all the higher excited states of appropriate symmetry is defined by *p*_1_ and *p*_2_ in the PJTE energy matrix elements *W*_*ΓΓ*_ and 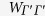
23:


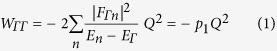



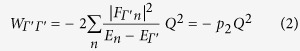


Then, the APES ε(*Q*) can be obtained from the secular equation of the perturbation theory:


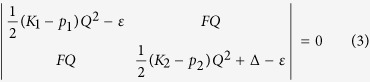


where *K*_1_, *K*_2_, *p*_1_ and *p*_2_ are the primary force constants as explained above, *F* is the PJT vibronic coupling constant between the two states, and Δ is the energy gap of the two coupled states.

The roots of this secular equation are:


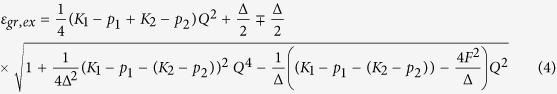


At small values of *Q* we get for the ground state:


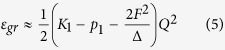


Then the condition of instability at *Q* = 0 of the ground state due to the PJT vibronic coupling is:


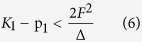


We thus separated all the excited states that destabilize the ground state via the PJTE in two parts: (1) all the higher states with small contributions taken into account by means of a second order perturbation correction *p* to the curvature *K*_0_, and (2) the lowest active excited state acting directly via the secular equation lowering the curvature by 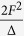. This means that if the chosen excited state is indeed the one responsible for the instability the numerical estimates should yield *K*_1_ − *p*_1_ > 0 and *K*_1_ − *p*_1_ − 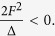


The direct *ab initio* calculation of these constants encounters difficulties (their calculation for some systems see in ref. 23). In the present paper we estimated them by fitting the solutions of the secular equation (4) to the *ab initio* data for the energy profiles of the corresponding states. For the *b*_2u_ distortion the PJTE two-level problem 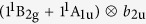 yields satisfactory results listed in Table 1. The numerical value of K_1_ − p_1_ − 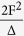 is −1.67, which satisfies the condition of instability (6); the contribution of all the other active excited states is included in the second-order perturbation correction p_1_. We checked the influence of the nearest one 2^1^A_1u_: its inclusion into the secular equation (7) below yields a very small contribution to the instability, thus justifying the two-level PJTE formulation for this case.

However, for the *b*_1g_ and *b*_2g_ instabilities only one active excited state does not yield positive *K*_1_ *−* *p*_1_ values, meaning higher excited electronic states should be included in the secular equation of direct PJT interaction. For a three-level problem the secular equation is:


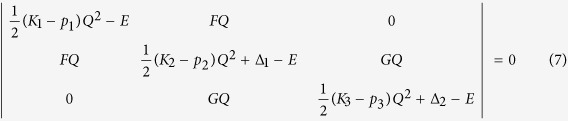


where in addition to the above denotations *K*_3_ is the primary force constant for the third term, *p*_3_ is the second order perturbation correction and *G* is the additional vibronic coupling constant to this term. Accordingly, the PJTE producing these distortions are 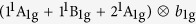 and 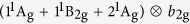, and the excited electronic states controlling these instabilities are 1^1^B_1g_ and 2^1^A_1g_ for the *b*_1g_ rhombic distortions, and 1^1^B_2g_ and 2^1^A_g_ for further *b*_2g_ puckering displacements in the rhombic configuration. The estimated PJTE constants are given in Table 1. The numerical results were evaluated by a fitting procedure with very small standard deviations of the residuals and the Pearson’s correlation coefficients equal to 1. The negative values of *K*–*p* in the excited states show that they are strongly influenced by the PJT coupling with higher electronic states.

### Extension to Si_4_R_4_ compounds with bulky substituents

The description of the origin of the structural features of Si_4_F_4_ as due to the PJTE may serve as a prototype for the investigation of Si_4_R_4_ analogues with more complicated substituents, such as the mentioned above Si_4_(EMind)_4_. However, the larger size systems possess many close-in-energy electronic states and normal modes (due to much lower molecular symmetry than D_4h_) which make a full analysis of the structural features in terms of symmetry adapted electronic states for the system as a whole much more difficult. In this case (as in many similar chemical problems) a more simple description can be achieved by considering the PJTE in terms of frontier molecular orbitals (MO) (obtained from the electronic structure calculations) which may happen to be localized in a much reduced “active center”, as in the case under consideration.

**Figure 5:**
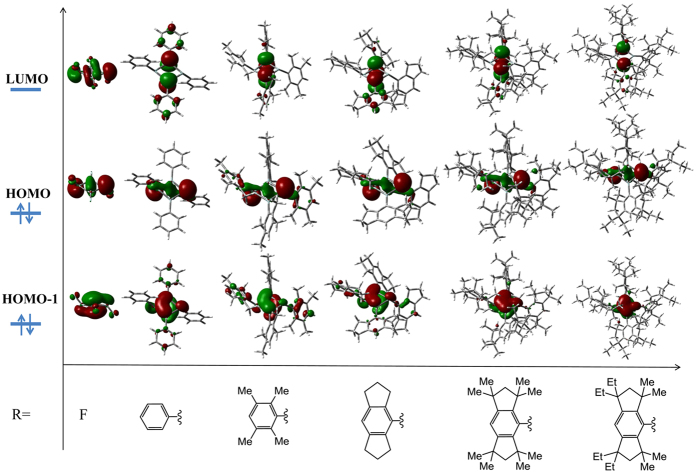
Calculated HOMO-1, HOMO, and LUMO of a series of trtrasilacyclobutadiene analogues Si_4_R_4_. The charge distributions are located at mostly the central fragment Si_4_ and the HOMO is essentially the same for all these compounds.

Figure 5 shows the highest occupied MO (HOMO), the next lower occupied MO (HOMO-1), and lowest unoccupied MO (LUMO) in the chair-like minima configurations of a series of Si_4_R_4_ with R as fluorine, phenyl, tetramethyl-phenyl, s-indacene, and EMind groups. We see that the electronic distributions of these frontier molecular orbitals are located mostly around the Si_4_ ring which is thus the active center of the whole molecule. In other words, the bulky substituents reduce the formal molecular point group, but play a minor role in the key electronic and vibronic properties of active center of Si_4_R_4_ that control their geometry via the PJTE. Therefore we can use the PJTE formulations and results obtained for Si_4_F_4_ with the D_4h_ high-symmetry configuration as a reference in exploring Si_4_R_4_ systems with bulky substituents. In the HOMO the electronic cloud is mainly on the σ bonding orbitals between two silicon atoms (to form the short diagonal), and non-bonding orbitals from the other two pyramidal silicon atoms (at the virtual long diagonal), with less charged density at the neighbor F or C atoms, and this HOMO electronic distribution is almost the same for all the Si_4_R_4_ systems.

**Figure 6:**
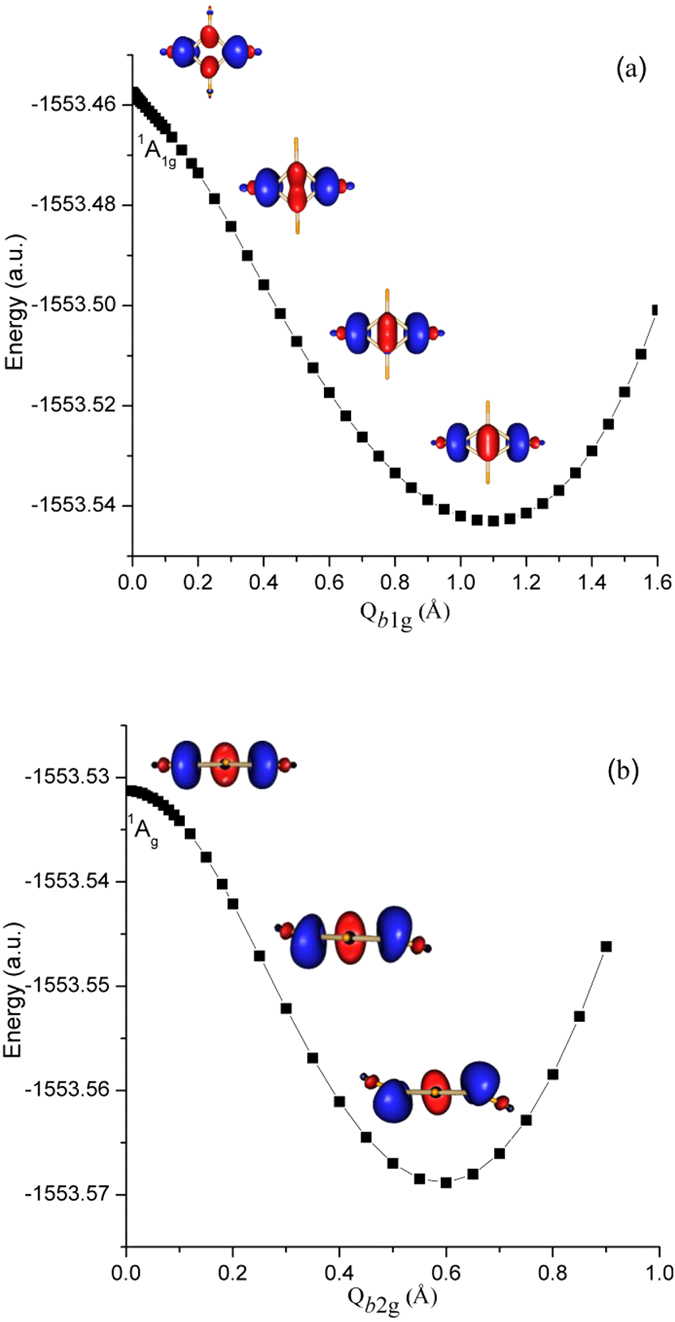
Tracing the HOMO of the Si_4_F_4_ molecule in the ^1^A_g_ ground state along first *b*_1g_ (**a**) and second *b*_2g_ (**b**) distortions. The HOMO actually originates from the empty *b*_1g_ orbital of the undistorted D_4h_ configuration. The graphic pictures in (**a**,**b**) are displayed in mutually perpendicular planes.

Based on the same Si_4_ ring active center and the same HOMO electronic distributions of the Si_4_R_4_ derivatives, we come to the prediction that they have the same kind of PJTE origin. By tracing this orbital distribution in the lowest ^1^A_g_ singlet electronic state along *b*_1g_ and *b*_2g_ distortions in Si_4_F_4_ (Fig. 6) we found that the HOMO actually originates from the empty *b*_1g_ MO of the undistorted D_4h_ configuration; it becomes *a*_g_ after distortion. As follows from the numerical data of the ab initio calculations illustrated in Fig. 6(a), the electronic distribution in this *b*_1g_ MO is mainly on the four σ*_Si−Si_ anti-bonding orbitals; under the *b*_1g_ distortion it gradually becomes bonding σ_Si−Si_ for two diagonal silicon atoms, and non-bonding MO for the other two silicon atoms. Then along the puckered *b*_2g_ mode the coplanar non-bonding MO of the latter turns to be located at the pyramidal positions, as shown in Fig. 6(b). In combination with the two-step distortions outlined above, we conclude that the initial *b*_1g_ MO in the undistorted high-symmetry configuration turns into the HOMO of Si_4_F_4_ in the chair-like structure. The changes in these charge distributions by distortions result from the admixture of excited states, and they take place spontaneously because this PJTE admixture of electronic states improves the bonding conditions and lowers the energy of the system by means of added covalency^17^,^18^. As expected and confirmed, the chair-like structures of Si_4_R_4_ compounds with bulky substituents R, including Si_4_(EMind)_4_, are qualitatively of the same PJTE origin as in the Si_4_F_4_ molecule.

### Structural correlations in Si_4_R_4_ compounds controlled by the PJTE

There are significant similarities and differences in the structural features of Si_4_R_4_ compounds with different R substituents. It follows from electronic structure calculations with geometry optimization^5^ that Si_4_H_4_ has only one equilibrium boat-like geometry, while Si_4_F_4_, as well as Si_4_Cl_4_ and Si_4_(OH)_4_ have both boat-like and chair-like minima, and the chair-like minimum is lower in energy. Since all these low-symmetry structures are controlled by the PJTE, their similarities and differences can be rationalized by employing the PJTE theory. We demonstrate this statement by comparisons among the mentioned four Si_4_R_4_ systems, Si_4_H_4_, Si_4_F_4_, Si_4_Cl_4_ and Si_4_(OH)_4_.

In the simplest two-level presentation of the PJTE (see Eqs. (3, 4, 5, 6)) the vibronic coupling of the electronic ground state of the system in the high-symmetry configuration to the excited state of appropriate symmetry makes the ground state unstable with respect to low-symmetry displacements if the condition of instability (6) is satisfied. The similarity in the substituents R (all of them from the second row except H) allows one to assume (based on previous experience^17^,^18^) that the energy gap is the main factor in the comparison of the possible instability of these four compounds.

The possible distortions of the high-symmetry configuration of these compounds, similar to the considered above Si_4_F_4_ case, in the two-level approximation is controlled by the PJTE problems 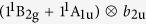, 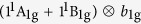, and 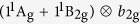 with energy gaps between the interacting electronic states Δ_1_, Δ_2_, and Δ_3_, respectively. The calculated values of the latter are given in Table 2 (Δ_3_ were calculated at the geometry of Q*b*_1g_ = 0.74Å). By comparing the Δ_1_ values, we see that they are very close, which indicates that the four molecules have almost equal possibility to form boat-like structures (which, however, are not necessarily ground state structures, see below). The situation is different with the Δ_2_ and Δ_3_ values. The Δ_2_ (0.70 eV) value in Si_4_H_4_ is approximately twice of those for Si_4_F_4_ (0.37 eV) and Si_4_(OH)_4_ (0.33 eV). It means that the PJT vibronic coupling along the *b*_1g_ and hence the possibility of generating a chair-like structure is much lower in Si_4_H_4_ as compared with the other three molecules. Furthermore, the smaller values of Δ_2_ and Δ_3_ compared with Δ_1_ in the Si_4_F_4_, Si_4_Cl_4_ and Si_4_(OH)_4_ molecules lead to a stronger PJTE coupling with *b*_1g_ and *b*_2g_ distortions than with *b*_2u_, explaining why the chair-like structures in these compounds are more stable than the boat-like ones. In this way the PJTE explains the origin of the main structural features of these Si_4_R_4_ compounds (R = H, F, Cl, OH), their similarities and differences, by comparing energy gaps to the active excited electronic states.

Similar correlations can be found between the structural features of Si_4_R_4_ compounds with larger substituents R and their electronic structure by comparing the energy gaps between PJT coupled molecular orbitals instead of electronic states. As shown above, in the D_4h_ configuration the electronic transition from the *e*_g_ (HOMO) to the *e*_u_ orbitals produces the ^1^A_1u_ excited electronic state which couples with ^1^B_2g_ ground state to form the boat-like structure, while the electronic transition from *e*_g_ to the empty *a*_1g_ or *b*_1g_ orbitals plays a key role in the formation of the chair-like structure. The calculated energy gaps of the *e*_u_, *a*_1g_ and *b*_1g_ empty orbitals relative to the *e*_g_ (HOMO) orbitals in the D_4h_ configuration denoted by δ_1_, δ_2_, and δ_3_, respectively, are given in Table 2. It is seen that the δ_1_ for all the four molecules are very close, but the δ_2_, δ_3_ values for Si_4_H_4_ are much larger than others, which also confirms that the chair-like structures are preferred in Si_4_F_4_, Si_4_Cl_4_ and Si_4_(OH)_4_, but not in Si_4_H_4_. In addition, the larger values of δ_2_, δ_3_ than δ_1_ in Si_4_R_4_ (R = F, Cl, OH) lead to the same conclusion as above that the chair-like structures are lower in energy than the boat-like structures.

### Other analogues: Si_4_(NX)_4_ and Ge_4_R_4_

**Figure 7:**
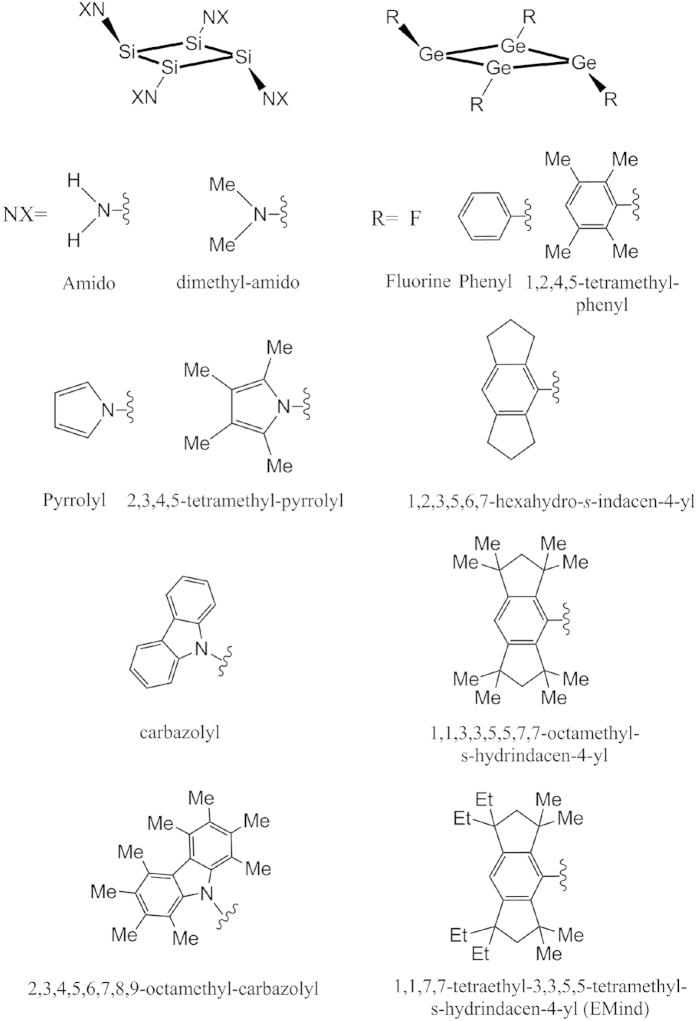
The stable chair-like structures of Si_4_(NX)_4_ and Ge_4_R_4_ compounds.

Below in this section we extend the results obtained above for tetrasilacyclobutadiene analogues Si_4_R_4_ with a characteristic series of substituents R (shown in Fig. 5) to include two other series, namely, Si_4_(NX)_4_ with amido substituents, and Ge_4_R_4_ which replaces silicon with the heavier germanium element. In the first of these two series, all the Si_4_(NX)_4_ analogues with substituents from NH_2_ to larger carbazolyl groups could stabilize in the chair-like structures shown in Fig. 7. Electronic structure calculations with geometry optimization starting with the puckered geometry converge either to the boat-like minimum with higher energy or back to the minimum with the chair-like structures. The latter is thus preferable in all tetrasilacyclobutadiene compounds except Si_4_H_4_. From the point view of stereoselectivity, the chair-like structure is also preferred because it lowers the steric hindrance induced by large substituents.

As shown above, in the series Si_4_R_4_ with R as fluorine, phenyl, tetramethyl-phenyl, s-indacene, and the EMind group, the origin of their chair-like structures are due to the PJTE. If the central Si_4_ ring is substituted by the Ge_4_, the chair-like structures are predicted to be stable too, as shown in Fig. 7. Calculated structural and electronic parameters for the Ge_4_R_4_ and Si_4_R_4_ series with the same substituents R are given in Table 3. The four-member ring in Ge_4_R_4_ and Si_4_R_4_ is planar and rhombic: the dihedral angle is very close to 0°, and the sum of the internal bond angles is very close to 360°, the bond length of the four Ge-Ge or Si-Si bonds are also very similar. As expected, the bond distances of the Ge-Ge bonds are longer than those of Si-Si bonds due to the bigger atomic radius of germanium than that of the silicon atom. Moreover, we found that the HOMO-LUMO gaps in the equilibrium configuration of most of Ge_4_R_4_ compounds are larger than those of Si_4_R_4_, especially in Ge_4_(EMind)_4_. The larger HOMO-LUMO gap *ceteris paribus* means the bigger hardness and higher stability with respect to external perturbations. Therefore, the Ge_4_R_4_ compounds with relatively large size substituents are predicted to have a stronger tendency to stabilize in the chair-like structures than the corresponding Si_4_R_4_ analogues. These predictions for Si_4_(NX)_4_ and Ge_4_R_4_ analogues may be useful in the search of potential precursors for semiconducting materials.

### Computational Methods

The geometry optimization and frequency calculations for the equilibrium structures of Si_4_R_4_ or Ge_4_R_4_ analogues are performed by the density functional theory with the B3LYP functional^34^,^35^ of the Gaussian 03 program^36^. Electronic excited states and potential energy profiles along different vibrational modes are obtained by the complete active space self-consistent field (CASSCF) method^37^,^38^ as implemented in MOLPRO 2010 packages^39^ based on the optimized geometries and normal coordinates of the ground state (^3^A_2g_) at the square configuration of D_4h_ symmetry by B3LYP method. The adiabatic potential energy surface in the lowest electronic state is obtained with B3LYP. The active space in the CASSCF method is composed of six electrons and all the valence empty orbitals, i.e., CAS(6,13) for Si_4_H_4_, Si_4_F_4_, and Si_4_Cl_4_, and CAS(6,17) for Si_4_(OH)_4_. The 6-31g(d,p) basis set is employed for all the calculations in this study.

## Conclusions

The PJTE is shown to be instrumental in revealing the main structural features of a series of tetrasilacyclobutadiene analogues, Si_4_R_4_ and Ge_4_R_4_, including large-size substituents, and rationalizing their similarities and differences. In all these compounds the excited electronic states that induce the deformation of the high-symmetry configuration in the ground state are determined, providing for a tool of possible manipulation of the structure (restoration of the planar configuration) by means of external perturbations. The formation of the boat-like structures originates from the PJTE vibronic coupling problem 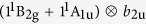 in the D_4h_ configuration, and the formation of chair-like structures is due to the PJT interaction of 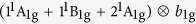 followed by the 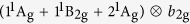. The qualitative analysis of the PJTE is enhanced by numerical estimates of the main vibronic coupling constants. Taking Si_4_H_4_, Si_4_F_4_, Si_4_Cl_4_ and Si_4_(OH)_4_ as examples, the substituent effect is analyzed, their structural differences shown to be due to the differences in the energy gaps to the PJT active excited states or between corresponding molecular orbitals. For Si_4_R_4_ with large substituents the same PJTE origin of preferred chair-like structures is deduced from their frontier molecular orbitals. For the Si_4_(NX)_4_ analogues with amido substituents and Ge_4_R_4_ homologs, the chair-like structures are still prefered. By comparison of the bonding character and HOMO-LUMO gaps in Ge_4_R_4_ and Si_4_R_4_ it is shown that the Ge_4_R_4_ compounds are expected to be more stable in the chair-like structures than the corresponding Si_4_R_4_ analogues, especially with the large-size substituents.

## Additional Information

**How to cite this article**: Liu, Y. *et al.* Geometry, Electronic Structure, and Pseudo Jahn-Teller Effect in Tetrasilacyclobutadiene Analogues. *Sci. Rep.* **6**, 23315; doi: 10.1038/srep23315 (2016).

## Supplementary Material

Supplementary Information

## Figures and Tables

**Figure 1:**
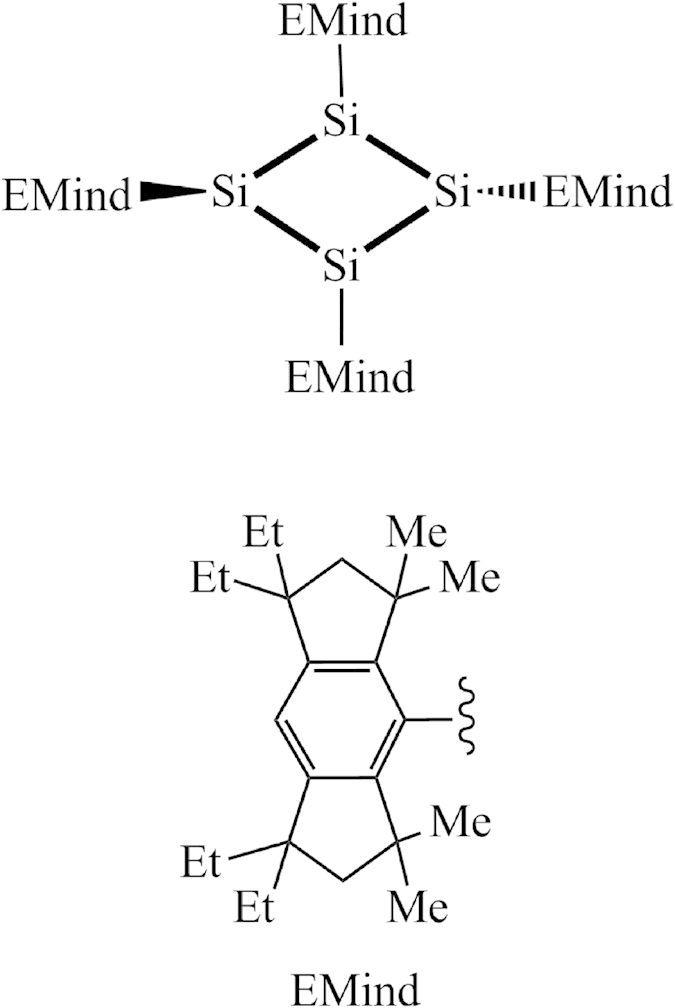
The 2-D structure of Si4(Emind)4 reported by Suzuki *et al.*^11^.

**Table 1:** The estimated parameters of the PJTE in Si_4_F_4_: primary force constants, linear vibronic coupling constants, and energy gaps to active excited states along *b*_2u_, *b*_1g_ and *b*_2g_ distortions.

Mode	*K*_1_−*p*_1_(eV/Å^2^)	*K*_2_−*p*_2_(eV/Å^2^)	*K*_3_−*p*_3_(eV/Å^2^)	*F* (eV/Å)	*G* (eV/Å)	Δ_1_(eV)	Δ_2_(eV)
*b*_2u_	2.51	−0.42		2.87		3.94	
*b*_1g_	0.27	0.43	−0.67	0.33	0.23	0.37	0.50
*b*_2g_	4.07	−13.98	19.29	3.83	3.74	1.50	2.36

**Table 2:** Energy gaps (in eV) between PJT interacting electronic states Δ_1_, Δ_2_, and Δ_3_, and PJT coupled molecular orbitals δ_1_, δ_2_, and δ_3_, in the PJTE formulations of the boat-like and chair-like structures of Si_4_H_4_, Si_4_F_4_, Si_4_Cl_4_, and Si_4_(OH)_4_ molecules.

Structure	PJTE	Energy gap	Si_4_H_4_	Si_4_F_4_	Si_4_Cl_4_	Si_4_(OH)_4_
Boat-like	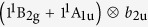	Δ_1_	4.02	3.94	4.19	3.81
		δ_1_	4.10	4.91	4.19	4.18
Chair-like	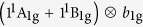 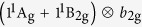	Δ_2_	0.70	0.37	0.51	0.33
		Δ_3_	1.41	1.50	1.02	1.30
		δ_2_	3.98	1.47	1.86	1.34
		δ_3_	3.71	1.38	1.51	1.23

**Table 3:** The HOMO-LUMO energy gaps ΔE (in eV) and bond distances d_Si−Si_, d_Ge−Ge_ (in Å) for the chair-like structures of Si_4_R_4_ and Ge_4_R_4_ compounds with different substituents R.

R	Si_4_R_4_		Ge_4_R_4_	
	ΔE	d_Si−Si_	ΔE	d_Ge−Ge_
Fluorine	3.74	2.309/2.309/2.309/2.309	3.21	2.458/2.458/2.458/2.458/
Phenyl	2.39	2.298/2.298/2.298/2.298	2.79	2.373/2.373/2.392/2.392
1,2,4,5-tetramethyl -phenyl	2.39	2.298/2.298/2.310/2.310	2.34	2.374/2.374/2.374/2.374/
1,2,3,5,6,7-hexahydro -s-indacen-4-yl	2.18	2.298/2.298/2.301/2.301	2.66	2.367/2.367/2.397/2.397/
1,1,3,3,5,5,7,7 -octamethyl- -s-hydrindacen-4-yl	1.87	2.313/2.313/2.328/2.328	2.02	2.369/2.369/2.376/2.376
EMind	1.86	2.311/2.315/2.323/2.331	2.11	2.370/2.371/2.376/2.379
