# Supplementary material for: Geometry, Electronic Structure, and Pseudo Jahn-Teller Effect in Tetrasilacyclobutadiene Analogues
Source: Sci Rep. 2016 Mar 21;6:23315. doi: 10.1038/srep23315 (PMC4800417; doi:10.1038/srep23315)

Supplementary Information

Geometry, Electronic Structure, and Pseudo Jahn-Teller Effect in Tetrasilacyclobutadiene Analogues

Yang Liu, Ya Wang, Isaac B. Bersuker

Supplementary data S1. The fully optimized chair-like and boat-like Si_4F_4 geometrical Cartesian coordinates (in Å) with B3LYP /6-31g (d,p) method.

(1) Chair-like minimum

Atom	x	y	z
Si	0.000000	0.000000	1.336953
Si	0.000000	1.882610	0.000000
Si	0.000000	0.000000	−1.336953
Si	0.000000	−1.882610	0.000000
F	0.000000	0.000000	2.951506
F	0.000000	0.000000	−2.951506
F	1.230062	2.948989	0.000000
F	−1.230062	−2.948989	0.000000

(2) Boat-like minimum

Atom	x	y	z
Si	0.000000	1.625262	0.326867
Si	1.625262	0.000000	−0.326867
Si	0.000000	−1.625262	0.326867
Si	−1.625262	0.000000	−0.326867
F	1.625262	0.000000	−1.955803
F	0.000000	1.625262	1.955803
F	−1.625262	0.000000	−1.955803
F	0.000000	−1.625262	1.955803

Supplementary Figure S2: The potential energy profiles of the ground and low-lying excited states of the Si_4F_4 molecule along b_{2u} distortion by including extra excited states between $^1A_{1u}$ and 2^1A_{1u} states. The terms of each curve are labeled at the left side of the figure in the order of energy creasing at Q=0.0.

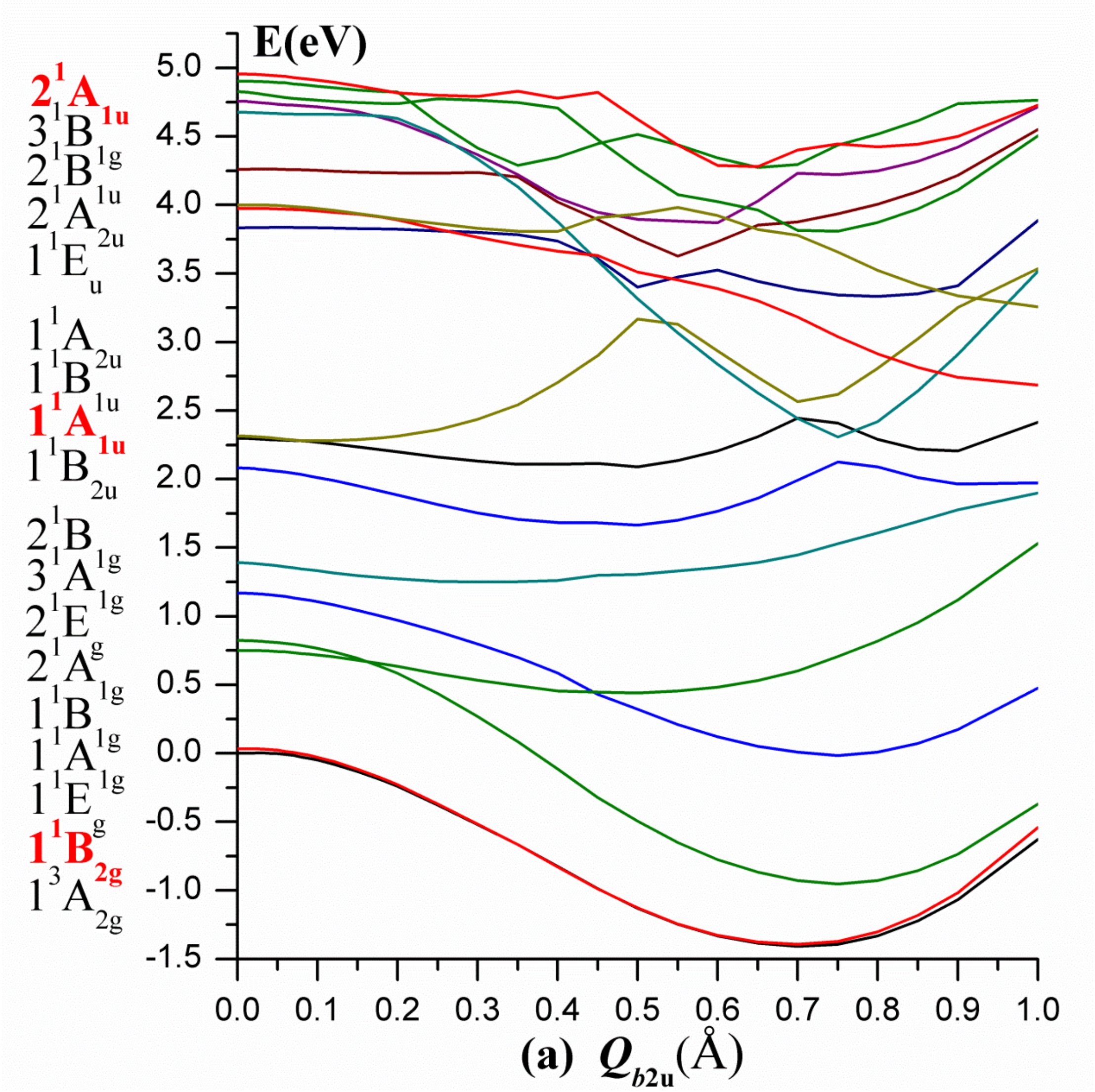

Supplement: Supplementary Information [file srep23315-s1.pdf]
